# Enhancing diabetes therapy adherence: a comprehensive study on glucometer usability for type 2 diabetes patients

**DOI:** 10.3389/fcdhc.2024.1328181

**Published:** 2024-05-14

**Authors:** Giovanni Toletti, Andrea Boaretto, Chiara Di Loreto, Riccardo Fornengo, Alfonso Gigante, Giovanni Perrone

**Affiliations:** ^1^ Department of Management, Economics and Industrial Engineering, Politecnico di Milano, Milan, Italy; ^2^ Personalive srl, Milan, Italy; ^3^ Unità Operativa Semplice Diabetologia - Distretto Del Perugino - Usl Umbria 1, Perugia, Italy; ^4^ Struttura Semplice Dipartimentale di Diabetologia e Malattie Metaboliche, Azienda Sanitaria Locale (ASL) TO4, Ospedale Civico di Chivasso, Chivasso, Italy; ^5^ Struttura complessa Diabetologia, Ospedale Cesare Zonchello, Nuoro, Italy; ^6^ Servizio Territoriale di Diabetologia Polo Sanitario Reggio Calabria Sud, Reggio Calabria, Italy

**Keywords:** self-monitoring of blood glucose (SMBG), diabetes, glucose monitoring, glucometer, usability, early adopters, device characteristics

## Abstract

**Background:**

Self-monitoring of blood glucose (SMBG) is a vital practice for type 2 diabetes (T2DM), and glucometers have the potential to improve therapy adherence. However, characteristics of glucometers improving their usability are underexplored. A knowledge gap exists regarding patients under 65, warranting further research for diabetes care improvement. Thus, this study aims to gather insights on glucometer accessibility, by analyzing the case of the Accu-Chek^®^ Instant glucometer by Roche Diabetes Care GmbH.

**Methods:**

Starting from a previous study having the objective of investigating devices’ features able to improve SMBG in over 65 T2DM patients, using the same device, we enlarged the scale, designing a survey that collected answers from 1145 patients of the Center and South of Italy, both under and over 65. 957 answers were analyzed, according to a threshold of 50% completion of the answers.

**Results:**

Our results show the major characteristics presented in Accu-Chek^®^ Instant are appreciated differently between patients under 65 and over 65, and between patients with or without previous experience with a glucometer.

**Discussions and conclusions:**

It emerged how Accu-Chek^®^ was perceived as more user-friendly among individuals under 65 compared to those aged 65 and over, where more people had prior experience, indicating how such a glucometer can be particularly helpful for naive patients. The study provides valuable insights to the academic discourse on glucometer features and their influence on therapy adherence.

## Introduction

A significant number of individuals with diabetes fail to attain their treatment objectives (1) primarily because they encounter obstacles in performing effective self-care. The practice of self-monitoring of blood glucose (SMBG) plays a pivotal role in safeguarding physical and mental well-being, especially for individuals suffering from type 2 diabetes mellitus (T2DM) (2). The appropriate use of glucometers can significantly enhance treatment adherence among T2DM patients, related clinical outcomes as well as quality of life (3).

While literature is proliferating about novel mHealth solutions for diabetes management and their design (4), the characteristics of glucometers improving their usability are barely investigated. Nevertheless, a substantial segment of T2DM patients contend with adverse perceptions pertaining to the management of their status (5).

A previous study investigated the physical and cognitive issues that over 65 years T2DM patients undergo when using glucometers for SMBG (6). From existing literature, it is evident that the primary physical issues patients face with healthcare devices include visual impairment and reduced tactile sensitivity (7, 8). As a result, devices or screens should not be too small (9, 10), and buttons should be metallic and it should be easy to understand if patients press them or not (10–12). Cognitive challenges such as memory deficits and decision-making difficulties can arise (13). To address them, devices should incorporate auditory or tactile features and enhance user-friendliness (14–16).

Two primary issues arise in self-monitoring of blood glucose (SMBG) practice: difficulty interpreting blood glucose (BG) results (17) and consequent lack of awareness regarding necessary actions, leading to reduced therapy adherence (18). The Target Range Indicator (TRI), utilizing a color spectrum, offers a solution by aiding in understanding and responding to BG results (17). Numerous studies have shown that the Color Range Indicator (CRI) improves diabetes management (18–22).

In the study mentioned before (6), authors investigated characteristics of devices supporting SMBG in patients over 65, interviewing 30 T2DM early adopters using the Accu-Chek^®^ Instant glucometer. This device features a backlit display, automatic strip ejector, wide dosing area, and CRI. Results showed enhanced user-friendliness with the backlit display and wide dosing area, while automatic strip ejection was deemed more hygienic. However, only half of the patients utilized and appreciated the CRI, with some unaware of its existence. Half found learning to use the glucometer easy, but some suggested healthcare personnel training. The pricking pen posed challenges due to toughened skin in elderly users. Attitudes toward BG results saved in the glucometer app varied based on technological literacy.

A recent RWE study investigated the degree of glycemic control by patients who used a blood glucose monitoring device linked to a mobile app through a Bluetooth connection. As a result, they denoted an improvement in glycemic monitoring thanks to the engagement with the app (23).

While that study focused only on patients aged 65 years and over, it is important to study also the opinions of patients aged under 65.

Indeed, extant literature has partially explored the results obtained from different age clusters. Patients from 16 years old were included in two studies (17, 18), patients from 30 to 70 years old were included in one study (19), patients aged more than 12 years old were considered in another study (21) and patients from 18 to 70 years old were involved in further research (22). However, there is still a lack of systematic assessment of the performance of the characteristics among those clusters, as well as scant evidence about how such characteristics are appreciated by different characteristics of the patients. Enhanced comprehension of the obstacles, requirements, and choices of people with T2DM is expected to enhance the development, execution, practical efficacy, and long-term adoption of such devices and consequently enhance clinical outcomes. Therefore, our aim is to conduct a survey to gather insights from patients with Type 2 Diabetes Mellitus (T2DM) regarding their experiences in managing glucose levels and the challenges they face in accessing, selecting, understanding, and optimally using diabetes technologies. We specifically focus on assessing the performance of the glucometer across various patient demographics. Starting from the explorative research conducted on over 65 patients (6), in this research we wanted to include also under 65 early-adopter patients, to evaluate the accessibility of the device. Our goal is to provide new insights into the accessibility of the device, particularly among patients who have encountered difficulties with previous devices and those who are new to using a glucometer. By validating these initial findings on a larger scale, we aim to enhance their reliability and applicability. Throughout this research, the Accu-Chek^®^ Instant glucometer produced by Roche Diabetes Care GmbH has been consistently utilized for data collection and analysis.

## Materials and methods

In order to examine the varying perceptions of glucometer features among diverse patient groups, the Accu-Chek^®^ Instant glucometer serves as an illustrative case study. This study further investigates the preliminary results of the usability of the glucometer Accu-Chek^®^ Instant, with a specific reference to the role of the above-mentioned characteristics. For this purpose, data about the experience with the device were widely collected from early adopters. Accordingly, a questionnaire was developed and submitted to 1145 patients in the Center-South of Italy. These patients have been identified thanks to the support of nurses in some hospitals. Patients with T2DM were provided with glucometers, and after one month, these patients were contacted by a third-party interviewer to schedule and carry out the interview. The interview was carried out through a phone call, so patients could answer properly to all the questions, being helped by the interviewer in providing relevant and appropriate answers. Eventually, all the data collected were cleaned and coded with ex-ante labels, so they could be analyzed and recurrency figures could be computed.

### Participants

Through the survey, 1145 answers were collected. Among the respondents, 957 answers were included in the analysis, as a threshold of 50% was set for the completion of the answers to be included. Participants were eligible if they were adults (aged ≥18 years) with T2CM, presently living in Italy, with current or prior experience with a glucometer device. Among all the collected answers, 749 (79%) people had prior experience and therefore their answers were analyzed.

The majority of the sample (55%) is represented by over 65 people, followed by people aged between 55-64 for 34%, while the remaining part of the sample is aged 45-54 (9%), 35-44 (2%), 25-34 (1%). The sample is well-balanced in terms of gender, as 56% of the respondents are male while females stand for 44%. Additionally, respondents come from regions in the Center and South of Italy. The data collection was geographically limited due to the fact that these regions have the highest concentration of early adopters. In **Table 1** the sample characteristics are summarized.

**Table 1:** Sample characteristics in terms of age, gender and origin.

Sample characteristics	
Age	55% over 65
	34% 55-64
	9% 45-54
	2% 35-44
	1% 25-34
Gender	56% male
	44% female
Origin	Center and South of Italy

### Survey content and design

The survey was divided into two parts: the first gathered socio-demographic and personal information about the patients, while the second focused on gathering insights into the most relevant themes regarding the technology used. The questions of the second part of the questionnaire was validated by a team of physicians. The initial part of the survey collected demographic and personal information such as age, gender, and location. Additionally, respondents were queried about their diagnosis, including diabetes type, treatment regimen, frequency of glycemic level measurement, and the presence of a caregiver, along with the caregiver’s identity. If a caregiver was assisting the patient in self-monitoring of blood glucose (SMBG), subsequent questions about glucometers were directed towards the caregiver, as they were considered the primary users of the device and could provide more pertinent insights into its actual usage. As a result, 94 interviews (10%) were conducted with caregivers.

Questions regarding the usability of glucometers focused on investigating their most common features. Respondents were asked about both the major problems and pain points, as well as strengths, related to their usage of previous glucometers. These questions allowed respondents to choose from options derived from the literature, including pricking fingers, the dosing area of the strip, reading or interpreting results, drawing blood, or reporting other pain points. Furthermore, respondents were asked if they experienced difficulty in handling the previous device overall, and if so, they were prompted to report the major limitations.

Subsequently, respondents were asked about their experience with Accu-Chek^®^ Instant. In this section, there was a higher focus on the diverse characteristics of the device and how those could influence its usability. First, they were asked if they could easily read results in terms of the size of both the display and the font. Subsequently, the arrow and colored scale were tested for their support in reading and interpreting more easily results and thus how they affect patients’ corrective behaviour. Two questions were related to the difficulties related to drawing blood and the impact of the wide dosing area and of the strip ejector on this action. Additionally, respondents were asked if they found Accu-Chek^®^ Instant easy to use with respect to prior glucometers. Respondents were eventually asked to pick the characteristics they like most about Accu-Chek^®^ Instant also considering those which have been majorly studied in past literature (17–23).

### Data analysis

Upon completion of the survey, the data underwent meticulous recording in a dedicated database. Prior to commencing the analysis, a rigorous data-cleaning process was conducted. Various analytical approaches were employed, including descriptive statistics such as frequency, percentage, and, where applicable, means, to compute all survey variables, particularly to elucidate the most common responses. Furthermore, an analysis was conducted to discern differences in responses between interviews conducted with patients and those with caregivers.

Subsequently, respondents were categorized into two clusters based on age, with one cluster comprising individuals under 65 and the other comprising those aged 65 and over. These analyses yielded pertinent insights into how diverse characteristics exhibited partially divergent results across the two age clusters, indicating varying roles in enhancing disease management based on patients’ age.

In the third phase of the analysis, attention was directed towards individuals who reported encountering difficulties with previous devices, with a comparative analysis of their responses concerning Accu-Chek^®^ Instant. The objective of this phase was to ascertain whether and to what extent the novel characteristics of the glucometer could alleviate some of the challenges patients encounter in executing SMBG.

The final phase of the analysis delved deeper into the differences in preferences between patients with prior experience and those utilizing a glucometer for the first time. This phase aimed to discern whether and how certain characteristics are beneficial for naïve adopters. The ensuing results of these analyses are delineated below.

## Results

In this section, the primary findings of the data analysis are presented. The first paragraph provides an overview of the descriptive analysis conducted. Subsequently, the second paragraph delves into the disparities observed between individuals aged under 65 and those aged 65 and above. The third paragraph entails an examination of patient experiences pertaining to previous devices encountered. Lastly, the fourth paragraph elucidates the contrasting responses between experienced and inexperienced patients. Additionally, we explored potential distinctions between responses provided directly by users and those communicated by caregivers on behalf of users. Nonetheless, no statistically significant differences were identified in this comparison.

### Descriptive analysis

The descriptive analysis encompasses both the analysis of the characteristics of the sample and of the experience with Accu-Chek^®^ Instant and previous glucometers.

For what concerns the sample description, most of the respondents have been diagnosed with T2DM for more than 5 years, while the remaining part of the sample has a diagnosis time between 1 and 5 years and less than one year. Check-up visits are planned by 89% of respondents once or twice per year while 10% plan examinations more than three times per year and only 1% check-up visits are scheduled with a frequency lower than once per year. Patients measure their glycemic level mostly once or twice a day, at least once a week or more than three times a day, while only around 2% measure only once a month. The vast majority is self-sufficient, and the remaining part is mostly helped by the son or daughter, the husband or wife or other relatives or caregivers. The pharmaceutical treatment was investigated too. Among the respondents to this question (86% of the sample answered), 80% are treated through oral and noninsulin injectable drugs, 22% are insulin-treated, 1% are based on oral secretagogues and 1% through lifestyle modification. In **Table 2**, the detailed percentages regarding disease management are reported.

**Table 2:** Disease Management approaches.

Disease management	
**Diagnosis time**	59% >5 Years
	26% 1< Years <5
	15% <1 Year
**Check-up visits**	10% > 3 times per year
	89% Once/twice per year
	1% < Once per year
**Glycemic level measurement**	49% Once/twice per day
	31% > Once per week
	18% > Three times per day
	2% Once per month
**Self-sufficiency**	89% Self-sufficient
	11% Supported by caregivers
**Pharmaceutical treatment**	80% Oral/Noninsulin injectable drugs
	22% Insulin-treated
	1% Oral secretagogues
	1% Lifestyle modification

We conducted an analysis of the prevalent characteristics among various glucometers, specifically focusing on how previous glucometers performed. Furthermore, we scrutinized participants’ opinions regarding previous glucometers and their experiences with the Accu-Chek^®^ Instant model.

The major problems respondents reported in handling previous glucometers are pricking fingers (42%), interpreting results (8%), drawing blood (8%), using the dosing area (7%), and reading results (5%). Although it is the most diffused problem, pricking fingers does not generate difficulties in 90% of respondents.

With respect to opinions related to Accu-Chek^®^ Instant, the vast majority found it very easy to use. The screen is easy to read for the majority of the sample, either as it is big enough (94%) or because the font size is easily readable (87%). The arrow and the colorimeter scale were highly appreciated by the vast majority of the respondents, as they eased the understanding of the results (65%) and eased the undertaking of corrective actions (62%). Additionally, the wide dosing area eases drawing blood and the automatic strip ejector makes the procedure more hygienic. In **Table 3** a summary of patients’ opinions related to Accu-Chek^®^ Instant is represented.

**Table 3:** Patients’ opinions related to Accu-Chek^®^ Instant.

	Non-favorable response	Neutral response	Favorable response
Accu-Chek^®^ Instant is easy to learn to use	7%	0%	93%
The screen is easy to read	1%	0%	99%
The arrow and the colorimetric scale make it easier and quicker to understand the results	4%	65%	31%
The arrow and the colorimetric scale make it easier the undertake of corrective actions	3%	35%	62%
The wide dosing area ease drawing blood	6%	25%	69%
The automatic strip ejector makes the procedure more hygienic	2%	16%	82%

Among the most appreciated device characteristics, the most voted ones are the wide screen (65%), ease of reading results (63%), its shape that makes it easy to handle (57%), the arrow and the colorimetric scale (42%) and the wide dosing area (37%).

### Results comparison based on age clusters

Respondents were categorized into two age groups: those under 65 and those aged 65 and over. This division aimed to discern notable disparities in the evaluation of characteristics between the clusters. Key insights were derived from this analysis, particularly regarding prior experiences with other glucometers. The majority of both under and over 65 had previously utilized other glucometers, with a higher percentage for the latter. Despite both groups indicating pricking fingers as the most discomforting activity, a notable disparity was observed. Specifically, 56% of individuals under 65 reported discomfort compared to 31% among those aged 65 and over. Additionally, among those under 65, drawing blood was the second most common discomfort, whereas interpreting results was reported as the second most frequent issue among those over 65. However, there are notable differences between the two age groups in their perceptions of certain glucometer characteristics. Individuals under 65 greatly appreciate the dosing area compared to those aged 65 and over, as it addresses one of their primary concerns.

Similarly, a higher percentage of respondents under 65 report difficulty in reading results compared to those over 65. Additionally, among individuals under 65, both the size of the screen and font were identified as facilitators for screen readability, while the arrow and colored scale were cited as aids for understanding results and taking corrective actions. Conversely, respondents aged 65 and over indicated somewhat different preferences. Although a majority acknowledged that the size of the screen and font contribute to screen readability, fewer respondents in this group found the arrow and colored scale helpful, for their capacity to assist in understanding results and undertaking corrective actions. Notably, a significant portion of respondents in both age groups reported not perceiving a difference in reading results through the arrow and colored scale.

In **Table 4** these data are summarized.

**Table 4:** Feedbacks comparison based on age clusters.

	Answers from under 65 patients	Answers from over 65 patients
Previous experience in the use of glucometers	79%	86%
Most uncomfortable feature with respect to previous experience	Pricking fingers (56%)	Pricking fingers (31%)
Second most uncomfortable feature with respect to previous experience	Drawing blood (13%)	Interpreting results (7%)
Appreciation of the dosing area	82%	60%
Reading results is one of the major difficulties	7%	4%
Characteristics that ease the reading of the screen	Size of the screen (95%); Font (89%)	Size of the screen (93%); Font (89%)
Arrow and colored scale as a support	To help in understanding results (75%); To help inundertaking corrective actions (72%)	To help in understanding results (57%); To help inundertaking corrective actions (54%)
Ease of use compared to previous experience	85%	70%

### Results comparison based on prior major problems

Building upon the findings of the previous analysis, which suggested that the appreciation of the novel characteristics of Accu-Chek^®^ Instant was not contingent upon age but rather on previous experience, a subsequent investigation was conducted to ascertain whether individuals who reported difficulties with prior glucometers experienced an improvement with Accu-Chek^®^ Instant.

For instance, out of the 56 respondents (7%) who reported encountering issues with using the strip for blood drop collection before transitioning to Accu-Chek^®^ Instant, 91% indicated that they no longer faced difficulties in collecting blood samples with Accu-Chek^®^ Instant. Additionally, 78% explicitly acknowledged the dosing area as significantly facilitating the blood sample collection process.

Similarly, among the 40 respondents (5%) who reported challenges in reading results, 97.5% stated that they encountered no issues with Accu-Chek^®^ Instant, attributing their ease in reading the device’s screen to its dimensions. Furthermore, 90% of these respondents affirmed the adequacy of the font size for facilitating result comprehension.

Furthermore, among the 66 respondents who identified interpreting results as a significant challenge, 95% reported finding the arrow and colored scale useful or very useful in comprehending results. Moreover, 93% of these individuals stated that these features aided in undertaking appropriate actions based on the results.

### Results comparison based on past experience

Regarding the characteristics of Accu-Chek^®^ Instant, notable differences emerged between the two groups. Experienced patients expressed appreciation for the screen dimensions (60%), ease of reading results (60%), and the device’s shape (53%). Conversely, for naïve patients, the same characteristics were valued, albeit with slight variations. Specifically, the most appreciated feature among naïve adopters was reading results (66%). However, both groups exhibited very similar levels of appreciation for screen dimensions (97% for naïve adopters and 98% for experienced adopters) and font size (91% for naïve adopters and 89% for experienced adopters) when it came to reading results. Similarly, Accu-Chek^®^ Instant shape is widely appreciated by naïve adopters (57%), as well as screen size (51%).

Additionally, the majority of inexperienced people (60%) think that the arrow and the colored scale are very or extremely useful for interpreting results, and all the naïve respondents think that they are useful for undertaking corrective actions.

## Discussion

We reported the results of a questionnaire shared with 1145 T2DM with past or current experience with glucometers. The objective of this shed novel light on the under-researched topic of the characteristics fostering the usability of such devices in order to increase adherence to the therapy. Indeed, prior studies have demonstrated a positive impact of structured SMBG on confidence in self-managing diabetes, which in turn leads to improvement in HbA1c (glycated haemoglobin) (24). Additionally, some evidence has shown that structured SMBG can provide psychosocial benefits to patients, fostering ongoing relevant debates about the most appropriate tools and methods (25). Therefore, it is paramount to discern the most suitable tools and their inherent characteristics for promoting Self-Monitoring of Blood Glucose (SMBG).

Therefore, this study deepens the preliminary insights gathered about the usability and accessibility of glucometers (6), engaging in the ongoing discourse, while incorporating perspectives from individuals under the age of 65 as well. The major focus of this paper is on the role characteristics have in increasing usability, especially in relation to some specific patients’ characteristics. From the first exploratory study, some characteristics emerged as particularly relevant for patients aged over 65, such as the automatic strip ejector and the backlit display, while some other characteristics remained with unclear opinions, such as the TRI. In this study, more clear results emerged.

The initial analysis revealed discernible differences between the two age cohorts. Specifically, a greater proportion of individuals under 65 reported discomfort in finger pricking compared to those aged 65 and over. This discrepancy may be attributed to the greater familiarity of this challenging task among older patients. Furthermore, while drawing blood emerged as the second most significant discomfort among those under 65, interpreting results ranked second among individuals aged 65 and over. This divergence could be reflective of the differing approaches to task engagement shaped by routine among the older demographic.

These disparities in discomfort experiences may influence the assessment of glucometer characteristics, which extends beyond patients aged 65 and over to encompass individuals under 65. For instance, the larger screen size is notably favored among the younger cohort compared to their older counterparts. Moreover, insights gleaned from the initial analysis, coupled with the initially reported challenges in result interpretation, suggest the utility of features such as arrows and colored scales for individuals grappling with result comprehension. However, as patients become more adept at interpreting results over time, the significance of these features diminishes.

Notably, Accu-Chek^®^ was perceived as more user-friendly among individuals under 65 (85%) compared to those aged 65 and over (70%).

Moreover, from the second set of analyses, our findings reveal that several features of these glucometers effectively address the primary challenges encountered by patients with previous glucometers. For example, the dosing area is widely praised among individuals who previously experienced difficulties in blood sample collection. Similarly, respondents who encountered challenges in result comprehension appreciated the screen and font size, while those struggling with result interpretation found the arrow and colored scale particularly helpful.

However, in the final step of the analysis, it became evident how these characteristics prove beneficial in addressing major issues even for naïve adopters. The findings indicate that Accu-Chek^®^ Instant facilitates the task of reading results for individuals who have no prior experience in this regard, as well as for those who compare Accu-Chek^®^ Instant with other glucometers based on their experience. Consequently, these results bolster the hypothesis that glucometer characteristics are advantageous not only for individuals aged 65 and over but also for a broader spectrum of patients, encompassing both inexperienced users and those encountering various difficulties in self-monitoring of blood glucose (SMBG). Hence, such a device should be targeted not solely at individuals aged 65 and over, but also at patients new to glucometers and those grappling with the aforementioned challenges.

This study contributes to the evolving academic discourse by investigating the features of glucometers that are predominantly favored by patients and have the potential to enhance their adherence to therapy. This study has several strengths, being a comprehensive assessment of glucometer characteristics and performing an analysis of the appreciation of such characteristics among different clusters of patients. Indeed, such study has generated further evidence in this field which can be exploited for generating proper recommendations on how to focus and leverage on these aspects to improve SMBG. This paper also contributes to the literature by evaluating its usability and compare it in patients aged over 65 in comparison to under 65 patients, differently from previous studies that had the goal to do a comparative analysis between Accu-Chek^®^ Instant and other glucometers. Indeed, while the literature shows evidence of over 65 patients (6), a comprehensive assessment of glucometer characteristics and its usability for a wider set of patients is still missing.

However, this study is subject to several limitations. Firstly, as we focused exclusively on the case of Accu-Chek^®^ Instant, certain minor features of other glucometers may have been overlooked, limiting the generalizability of our findings. Additionally, our study captured results at a specific point in time, potentially overlooking any evolving perceptions over time. Therefore, future research could explore how perceptions change after several months from initial usage, providing further insights. Moreover, a significant limitation pertains to the selected sample, which was confined to the centre and south of Italy. Consequently, expanding the sample to encompass other regions within Italy and even extending the study to include participants from other countries would enhance the generalizability and robustness of our findings.

## Conclusions

The conclusion of this study underscores the pivotal role of glucometers in facilitating self-monitoring of blood glucose (SMBG) among Type 2 Diabetes Mellitus (T2DM) patients, where therapy adherence is significantly influenced by the characteristics and usability of these devices. Through a comprehensive survey encompassing 957 responses from both under and over 65 early adopter patients, this paper aimed to elucidate the features of glucometers capable of enhancing their usability.

Overall, the findings offer valuable insights into the target patients for the Accu-Chek^®^ Instant glucometer. However, these insights are not limited to this specific device and could be extrapolated to inform the design and usage of other glucometers. The study highlights characteristics appreciated by both under and over 65 early adopter patients, shedding light on their preferences compared to patients with previous experience using glucometers.

Consequently, this study serves as a foundational resource, providing practical guidelines for healthcare professionals involved in diabetes management. As suggested in the Discussion section, future research endeavors could incorporate additional regions within Italy to offer a comprehensive nationwide perspective. Moreover, exploring this topic in other countries could reveal potential cross-cultural variations. Finally, replication of the study with the same population in the future could elucidate any changes in responses over time as patients gain experience.

## Data availability statement

The raw data supporting the conclusions of this article will be made available by the authors, without undue reservation.

## Ethics statement

Written informed consent was obtained from the individual(s) for the publication of any potentially identifiable images or data included in this article.

## Author contributions

GT: Writing – original draft, Writing – review & editing, Conceptualization, Investigation, Methodology. AB: Writing – original draft, Writing – review & editing, Conceptualization, Formal analysis, Investigation, Methodology. CD: Validation, Writing – review & editing. RF: Validation, Writing – review & editing. AG: Validation, Writing – review & editing. GP: Validation, Writing – review & editing.

## References

[1] Chen Y Rolka D Xie H Saydah S. Imputed state-level prevalence of achieving goals to prevent complications of diabetes in adults with self-reported diabetes — United States, 2017–2018. MMWR Morb Mortal Wkly Rep. (2020) 69:1665–70. doi: 10.15585/mmwr.mm6945a1 PMC7660663 33180755

[2] Chen HM Su BY. Factors related to the continuity of care and self-management of patients with type 2 diabetes mellitus: A cross-sectional study in Taiwan. Healthcare. (2022) 10:1–12. doi: 10.3390/healthcare10102088 PMC9602078 36292535

[3] Greenwood DA Grady M. Healthcare professional perceptions of blood glucose meter features that support achievement of self-management goals recommended by clinical practice guidelines. J. Diabetes Sci. Technol. (2020) 15:1142–52. doi: 10.1177/1932296820946112 PMC8411481 32772855

[4] Rasche P Mertens A Miron-Shatz T Berzon C Schlick CM Jahn M. Seamless recording of glucometer measurements among older experienced diabetic patients – A study of perception and usability. PloS One. (2018) 13:1–10. doi: 10.1371/journal.pone.0197455 PMC5969745 29799861

[5] Lin M Chen T Fan G. Current status and influential factors associated with adherence to self-monitoring of blood glucose with type 2 diabetes mellitus patients in grassroots communities: a cross-sectional survey based on information-motivation-behavior skills model in China. Front. Endocrinol. (Lausanne). (2023) 14:1–13. doi: 10.3389/fendo.2023.1111565 PMC10335788 37441499

[6] Pinelli M Lettieri E Boaretto A Casile C Citro G Zazzaro B. Glucometer usability for 65+ Type 2 diabetes patients: insights on physical and cognitive issues. Sensors. (2022) 22:1–10. doi: 10.3390/s22166202 PMC9416294 36015970

[7] Omori M Watanabe T Takai J Takada H Miyao M. Visibility and characteristics of the mobile phones for elderly people. Behav. Inf Technol. (2002) 21:313–6. doi: 10.1080/0144929021000048466

[8] Chang JJ Hildayah Binti Zahari NS Chew YH. The design of social media mobile application interface for the elderly. 2018 IEEE Conf Open Syst. ICOS. (2018) 2019:104–8. doi: 10.1109/ICOS.2018.8632701

[9] Lee JH Kim YM Rhiu I Yun MH. A persona-based approach for identifying accessibility issues in elderly and disabled users’ interaction with home appliances. Appl. Sci. (2021) 11:1–28. doi: 10.3390/app11010368

[10] Kurniawan S Mahmud M Nugroho Y. A study of the use of mobile phones by older persons. Conf Hum. Factors Comput. Syst. - Proc. (2006), 989–94. doi: 10.1145/1125451

[11] Irie T Matsunaga K Nagano Y. Universal design activities for mobile phone: Raku Raku PHONE. Fujitsu Sci. Tech J. (2005) 41:78–85.

[12] Adams N Stubbs D Woods V. Psychological barriers to Internet usage among older adults in the UK. Med. Inform Internet Med. (2005) 30:3–17. doi: 10.1080/14639230500066876 16036626

[13] Audu O Van Benthem K Herdman CM. Validation of virtual reality cognitive assessment for pilots across the lifespan. In: LNAI, Lecture Notes in Computer Science (including subseries Lecture Notes in Artificial Intelligence and Lecture Notes in Bioinformatics) (Springer: Cham), vol. Vol. 12767. (2021). p. 3–18. doi: 10.1007/978-3-030-77932-0_1

[14] Curreri C Trevisan C Carrer P Facchini S Giantin V Maggi S. Difficulties with fine motor skills and cognitive impairment in an elderly population: the Progetto Veneto Anziani. J. Am. Geriatr. Soc. (2018) 66:350–6. doi: 10.1111/jgs.15209 29322488

[15] Shi A Huo F Han D. Role of interface design: A comparison of different online learning system designs. Front. Psychol. (2021) 12:1–9. doi: 10.3389/fpsyg.2021.681756 PMC8438291 34531786

[16] Wang SF. Research on web interface barrier-free design for elderly people. Proc. - 2020 Int. Conf Intell. Des. ICID 2020. (2020) Icid):157–9. doi: 10.1109/ICID52250.2020

[17] Grady M Cameron H Katz LB. Patients with diabetes using a new glucose meter with blood sugar mentor and dynamic color range indicator features show improved interpretation and willingness to act on blood glucose results (ASCEND study). J. Diabetes Sci. Technol. (2020) 15:1168–76. doi: 10.1177/1932296820949873 PMC8411471 32830523

[18] Grady M Katz LB Strunk CS Cameron H Levy BL. Examining the impact of a novel blood glucose monitor with color range indicator on decision-making in patients with type 1 and type 2 diabetes and its association with patient numeracy level. JMIR Diabetes. (2017) 2. doi: 10.2196/diabetes.8299 PMC6238893 30291065

[19] Al Hayek A Alwin Robert A Al Dawish M. Patient satisfaction and clinical efficacy of novel blood glucose meters featuring color range indicators in patients with type 2 diabetes: A prospective study. Cureus. (2020) 12:1–8. doi: 10.7759/cureus.11195 PMC7704060 33269126

[20] Drincic AT. Analysis of “Use of blood glucose meters featuring color range indicators improves glycemic control and patients with diabetes in comparison to blood glucose meters without color (ACCENTS study). ” J. Diabetes Sci. Technol. (2018) 12:1220–2. doi: 10.1177/1932296818793115 PMC6232752 30095006

[21] Katz LB Grady M Stewart L Cameron H Anderson PA Desai A. Patient and health-care professional satisfaction with a new, simple, high accuracy blood glucose meter with color range indicator. Indian J. Endocrinol. Metab. (2017) 21:322–8. doi: 10.4103/2230-8210.202030 PMC5367238 28459033

[22] Grady M Katz LB Levy BL. Use of blood glucose meters featuring color range indicators improves glycemic control in patients with diabetes in comparison to blood glucose meters without color (ACCENTS study). J. Diabetes Sci. Technol. (2018) 12:1211–9. doi: 10.1177/1932296818775755 PMC6232745 29848106

[23] Grady M Cameron H Bhatiker A Holt E Schnell O. Real-world evidence of improved glycemic control in people with diabetes using a bluetooth-connected blood glucose meter with a mobile diabetes management app. Diabetes Technol. Ther. (2022) 24:770–8. doi: 10.1089/dia.2022.0134 PMC9529309 35653730

[24] Holmes-Truscott E Baptista S Ling M Collins E Ekinci E Furler J. The impact of structured self-monitoring of blood glucose on clinical, behavioral, and psychosocial outcomes among adults with non-insulin-treated type 2 diabetes: a systematic review and meta-analysis. Front. Clin. Diabetes Healthc. (2023) 4:1–12. doi: 10.3389/fcdhc.2023.1177030 PMC10157033 37153750

[25] Babbott KM Serlachius A. Developing digital mental health tools for youth with diabetes: an agenda for future research. Front. Clin. Diabetes Healthc. (2023) 4:1–5. doi: 10.3389/fcdhc.2023.1227332 PMC10367007 37497385

